# The relationship between antithrombin administration and inflammation during veno-venous ECMO

**DOI:** 10.1038/s41598-022-17227-7

**Published:** 2022-08-22

**Authors:** Mauro Panigada, Elena Spinelli, Stefano De Falco, Dario Consonni, Cristina Novembrino, Massimo Boscolo Anzoletti, Giovanna Panarello, Giovanna Occhipinti, Claudia C. dos Santos, Antonio Pesenti, Antonio Arcadipane, Giacomo Grasselli

**Affiliations:** 1 Department of Anaesthesiology, Critical Care and Emergency, Intensive Care and Emergency, Fondazione IRCCS Ca’ Granda Ospedale Maggiore Policlinico, Milan, Italy; 2 Epidemiology Unit, Fondazione IRCCS Ca’ Granda Ospedale Maggiore Policlinico, Milan, Italy; 3 Clinical Laboratory, Fondazione IRCCS Ca’ Granda Ospedale Maggiore Policlinico, Milan, Italy; 4 Department of Anesthesiology and Intensive Care, ISMETT IRCCS (Istituto Mediterraneo per i Trapianti e Terapie ad Alta Specializzazione), UPMC, Palermo, Italy; 5 Keenan Research Centre for Biomedical Science, St Michael’s Hospital, Unity Health Toronto, Toronto, ON Canada; 6 Department of Pathophysiology and Transplantation, University of Milan, Milan, Italy

**Keywords:** Clinical trials, Randomized controlled trials, Respiratory distress syndrome

## Abstract

Veno-venous Extracorporeal Membrane Oxygenation (ECMO) is used in the most severe cases of respiratory failure and further exacerbates the patients’ inflammatory status. Antithrombin is supplemented during ECMO for its anticoagulant effects, but it also deploys anti-inflammatory properties. In this pre-specified ancillary study of the GATRA trial [NCT03208270] we aimed to evaluate the relationship between antithrombin and inflammation during ECMO. Forty-six patients were included in the study, 23 were randomized to receive antithrombin to maintain a level of 80–120% (study group) and 23 were randomized not to be supplemented (control group). Anticoagulation was provided in both groups with heparin infusion. Six cytokines were measured at 5 timepoints from prior to ECMO start to 7 days after ECMO removal. Cytokines decreased during the study but overall were not very different in the two groups. Testing the interaction between the study group and timepoints suggests that the administration of antithrombin led to a more rapid decrease over time of IL-6, IL-1β, TNF-⍺ and Pro-ADM. Plasma levels of antithrombin (either endogenous or exogenous) were negatively associated with all cytokines. Inflammation decreases during ECMO but a causal effect of antithrombin administration on the reduction of inflammation (and its clinical relevance) must be confirmed by appropriately powered studies.

## Introduction

Veno-venous Extracorporeal Membrane Oxygenation (ECMO) has emerged as a therapeutic option for patients with the most severe forms of acute respiratory failure when conventional and protective mechanical ventilation fails to provide adequate organ support. Despite major improvements in pump and circuit design and biocompatibility^1^, exposure to the non-endothelialized surface of the artificial circuit^2^ still results in enhanced coagulopathy and inflammation characterized by activation of components of the innate immune system such as complement, endothelial cells, leukocytes and platelets^3^. Plasma levels of several pro-inflammatory cytokines rise rapidly after ECMO initiation^4–6^, and may initiate, exacerbate, and/or propagate underlying endothelial and microcirculatory injury^7^. During extracorporeal therapies, unfractionated heparin is commonly used to avoid activation of the coagulation cascade and clinical efficacy depends on attaining adequate anticoagulation.

Antithrombin, a circulating protein that inactivates thrombin and other coagulation factors, is essential to the therapeutic effects of heparin and is often supplemented during ECMO^8^. In addition to its serine-protease inhibitor anti-coagulant role^9^, AT also has anti-microbial and inflammatory properties^10^. Antithrombin can bind to and perforate bacterial cell walls. Peptide fragments derived from proteolytic degradation of antithrombin exert antibacterial effects. Antithrombin binds to structures on the glycocalyx (syndecan-4) and modulates interactions with cell surface receptors to ultimately attenuate cellular activation causing increased release of prostacyclin that inhibits aggregation and activation of platelets^11^, decreased leukocyte rolling and adhesion^12,13^ and inhibition of proinflammatory cytokines expression by the endothelial cells^14–16^.

We recently published a clinical trial which investigated the effect of antithrombin administration on heparin anticoagulation during veno-venous ECMO^17^. Administration of antithrombin to target plasmatic activity between 80 and 120% did not result in decreased heparin dose compared to the control group which did not receive antithrombin supplementation. An ancillary aim of the study was to assess the effect of antithrombin administration on the inflammatory response during ECMO. The aims of the present study are: (1) to evaluate whether antithrombin administration during veno-venous ECMO for respiratory failure results in a reduction of circulating levels of pro-inflammatory mediators in plasma, and (2) to determine whether the plasmatic activity of antithrombin correlates with inflammation.

## Methods

The present study is a pre-specified^18^ ancillary study of the “Randomized Controlled Trial of Antithrombin Supplementation During Extracorporeal Membrane Oxygenation”^17^. It was approved by the local Institutional Review Boards, Milan Area B Ethics Committee (ref. approval no. 191_2017bis), authorized by the Italian Drug Agency (EudraCT 2016-004534-23) and registered at ClinicalTrials.gov (NCT03208270, registered on 05/07/2017). All methods were performed in accordance with the relevant guidelines and regulations, full protocol is available as open source publication. Written informed consent was obtained according to Italian regulation. The main study was an investigator-initiated, randomized, single-blind, two-arm trial conducted in the ICUs of two Italian referral ECMO centers (Fondazione IRCCS Ca’ Granda—Ospedale Maggiore Policlinico, Milano; Istituto Mediterraneo per i Trapianti e Terapie ad Alta Specializzazione [ISMETT], Palermo) to compare the administration of antithrombin concentrate to maintain a plasmatic level 80–120% (treatment) or not (control) on the total amount of heparin required to maintain an activated partial thromboplastin time (aPTT) of 1.5–2.0 in adult patients requiring veno-venous ECMO for severe respiratory failure. Exclusion criteria were: age less than 18 years, veno-arterial ECMO, or a decision to withhold heparin because of a history of induced thrombocytopenia or a perceived very high risk of bleeding (e.g., after major surgery). The configuration of the ECMO support and the algorithm to standardize monitoring and circuit change criteria have been described in the main study^16^. Antithrombin levels were measured before ECMO start and then once per day in both groups, by using an assay based on a synthetic chromogenic substrate and factor Xa inactivation (Hemosil Liquid Antithrombin; Werfen). The method is not influenced by Heparin Cofactor II and by heparin up to 2 U/mL. Antithrombin concentrate was administered as an extended infusion^19^. Supplementation of antithrombin and anticoagulation with heparin was protocolized as previously described^17^. The study was interrupted if the patient died or developed a condition contraindicating further heparin use (i.e., the onset of heparin-induced thrombocytopenia or active bleeding). Patients were observed until ICU discharge or death.

### Study patients

Patients were eligible for this study if they had available stored plasma samples for cytokines measurements. Samples from two patients (one in the control and one in the treatment group, both randomized at ISMETT) were not available, leaving 46 (out of a total of 48 randomized in the main study) patients for analysis (23 in the control and treatment group, respectively).

Patients at Fondazione IRCCS Ca’ Granda (n = 20) received Epsoclar (Pfizer Italia SrL, Latina, Italy) as heparin preparation and ATKED (Kedrion SpA Castelvecchio Pascoli, Barga [LU], Italy) as antithrombin concentrate. Patients at ISMETT (n = 3) received Pharepa (Pharmatex Italia Srl, Milan, Italy) as heparin preparation and Ambinex (Grifols SA, Barcelona, Spain) as antithrombin concentrate.

### Data collection and transfusion protocol

Demographic characteristics, causes of respiratory failure, disease severity scores including Sepsis-related Organ Failure Assessment (SOFA), Simplified Acute Physiology Score (SAPS) and Disseminated Intravascular Coagulation Score^20^ were collected. Complete blood count and coagulation variables including prothrombin time ratio, aPTT ratio, fibrinogen, and d-dimer levels were measured at least three times per day. Protein C and protein S were measured at baseline. Antithrombin and C-reactive protein were measured once per day in all patients.

In both groups, packed red blood cells (RBCs), platelets, and fresh frozen plasma or fibrinogen concentrate were transfused to maintain hemoglobin concentration greater than 10 g/dL, platelet count greater than 50,000/mm3, and fibrinogen level greater than 150 mg/dL.

### Timing of blood sampling for cytokines measurement

Four aliquots of plasma, 0.5 mL each, were obtained from each patient, by centrifugation of ethylene-diamine-tetra-acetic acid (EDTA) blood at 3000 revolutions per minute (RPM) for 15 min at 4 °C, prior to ECMO start, 24 h after ECMO start, 72 h after ECMO start, before ECMO removal, and 7 days after ECMO removal or before discharge from the ICU whichever happened first. Aliquots were immediately stored after collection at − 80 °C. In the case the study was interrupted the penultimate sample was considered the last sampling point.

Cytokines levels were measured in duplicate at the central laboratory of Fondazione IRCCS Ca’ Granda on EDTA-plasma samples by enzyme-linked immunoadsorbent assays. In particular, IL-8 and IL-10 were measured using commercial kits (BioVendor, Brno, Czech Republic)^21^, being the analytical ranges 1.5–100 pg/mL and 3–200 pg/mL, respectively and the inter-assay coefficient of variation (CV%) < 8.5% for IL-8 and < 4.9% for IL-10. For the determination of IL-6, IL-1β and TNF-α specific kits (Cayman Chemical, Ann Arbor, MI, USA)^22^ were used with analytical range 3.9–250 pg/mL; inter-assay imprecision was 5.4–13.3% for IL-6, 2.3–11.4% for IL-1β and 9–10.4% for TNF-α. Samples with a concentration higher than the respective analytical range were properly pre-diluted. Levels of pro-adrenomedullin (ADM) were assessed by a commercial fluoroimmunoassay (BRAHMS MR-proANP KRYPTOR; BRAHMS GmbH, Hennigsdorf, Germany): the lower detection limit of the assay was 0.05 nmol/L; the inter-assay CV ranged from 5.6% for high concentrations (> 10 nmol/L) and 17.5% for low levels (0.2–0.5 nmol/L).

### Study endpoints

The endpoints of the study were: (1) Plasma levels of cytokines in the two groups during the study and (2) the strength of the correlation between the plasmatic activity of antithrombin and plasma levels of cytokines.

### Statistical analysis

The sample size was calculated for the main study^17^ to detect a reduction in heparin consumption in patients who receive antithrombin supplementation.

Shapiro–Wilk test was used to assess normal distribution of continuous variables. As most of the variables presented a non-normal distribution, we reported median and 25th–75th percentiles or geometric means as appropriate. Categorical variables are presented as frequency and percentages.

We compared quantitative and categorical variables at enrollment between the study groups with Wilcoxon rank-sum and chi square tests, respectively.

To evaluate the effect of antithrombin administration on cytokines between the two study groups we used random-intercept linear models on log-transformed outcomes (cytokines) to account for repeated outcome (timepoints during the study). Time (timepoints) was treated as a quantitative variable (i.e., we assigned values from 0 to 4 to the five timepoints) assuming a constant effect of antithrombin along timepoints. All models were adjusted for basal value of the dependent variable (cytokines) because patient in the control group had higher levels of cytokines before the beginning of the study.

An interaction (product) term between time (timepoints) and the study groups was also added and Wald test was used to evaluate a time-varying effect of antithrombin between the two study groups (i.e., to test if time trends in the two groups were parallel). When interaction was significant (p < 0.05. i.e., there were non-parallel trends between the two groups) the interaction term was kept in the model. The interaction coefficient was expressed as change (%) per each time-point with 95% confidence intervals (CI). Sensitivity analyses were also conducted by treating time as a categorical variable and including four product terms (tested with a global Wald test) in the models.

To evaluate the association between the plasmatic activity of antithrombin and log-transformed cytokines (all timepoints analyzed together), the Pearson’s correlation coefficients and the regression slopes (again expressed as percent change) from random-intercept linear regression models were calculated. To evaluate whether the antithrombin administration modified the association between plasmatic activity of antithrombin and cytokines, an interaction term between the study groups and the plasmatic activity of antithrombin was added to the models. All analyses were performed using Stata 17.0 (StataCorp, College Station, TX, USA).

## Results

This study included 46 patients, of which 41 were randomized in Milan (20 in the treatment group) and 5 in Palermo (3 in the treatment group). All patients in the treatment group received antithrombin according to the study design (median 3000 (3000–2000) IU/day); none of the patients in the control group received antithrombin.

The two study groups had similar baseline characteristics (Table 1). Respiratory failure was due to acute respiratory distress syndrome (ARDS) in 40 (87%) patients and in 6 (13%) cases to chronic obstructive pulmonary disease (COPD) exacerbation. Viral pneumonia was the most frequent causative agents of ARDS in both groups. Among baseline coagulation parameters, platelets, prothrombin time (PT) and aPTT, natural anticoagulants (Protein C and Protein S) and antithrombin were not consumed and slightly higher (although not significant) in the control group. D-Dimer was elevated in both groups. Only a minority of patients (2 and 3 patients in the control and study group respectively) had disseminated intravascular coagulation at enrollment. The severity scores SOFA and SAPSII were not different in the two groups.

**Table 1:** Baseline characteristics of the patients.

	Control	N	Treatment	N	P value*
Age (years)	59.0 (45–63)	23	57.0 (47–61)	23	0.61
Female sex (N, %)	8 (34.8)	23	6 (26.1)	23	0.75
BMI (kg/m^2^)	28.1 (26.2–30.9)	23	27.3 (23.1–32.7)	23	0.71
**Cause of respiratory failure (N, %)**		23		23	0.64
Extrapulmonary ARDS	2 (8.7)		1 (4.3)		
ARDS of unknown origin	1 (4.3)		2 (8.7)		
Cryptogenic organizing pneumonia	1 (4.3)		0 (0)		
Aspiration pneumonia	0 (0)		1 (4.3)		
Pulmonary ARDS (bacterial pneumonia)	5 (21.7)		9 (39.1)		
Exacerbation of COPD	2 (8.7)		2 (8.7)		
Status asthmaticus	2 (8.7)		0 (0)		
Pulmonary ARDS (viral pneumonia)	10 (43.5)		8 (34.8)		
**Baseline laboratory parameters**					
Antithrombin (%)	92 (51–116)	21	80 (48–105)	21	0.49
Hemoglobin (g/dL)	10.3 (9.0–12.1)	23	11.7 (12.1–13.1)	23	0.08
White blood cells (10^3^/mmc)	9.9 (5.7–19)	23	10.0 (7.9–17.7)	23	0.60
Platelets (10^3^/mmc)	177.0 (130.0–268.0)	23	181.0 (111.0–256.0)	23	0.96
Fibrinogen (mg/dL)	524.0 (425.0–673.0)	23	560.0 (432.0–770.0)	23	0.64
PT (ratio)	1.1 (1.0–1.2)	23	1.2 (1.0–1.4)	23	0.13
aPTT (ratio)	1.0 (0.9–1.1)	19	1.1 (1.0–1.3)	15	0.03
D-dimer (mg/dL)	4449.0 (2510.0–6413.0)	23	3167.0 (1901.0–5996.0)	23	0.74
CRP (mg/dL)	14.4 (10.3–24.0)	21	19.0 (12.6–23.8)	21	0.58
Protein C	101.0 (53.0–125.0)	21	83.5 (55.0–96.0)	18	0.08
Protein S	81.0 (70.0–114.0)	21	73.0 (58.0–82.0)	18	0.10
DIC (N, %)	2 (8.7)	23	3 (13.0)	23	1.0
DIC score**	2.0 (2.0–4.0)	23	3.0 (2.0–4.0)	23	0.13
SOFA	8.0 (5.0–11.0)	23	9.0 (5.0–16.0)	23	0.41
SAPS II	36.0 (29.0–51.0)	23	47.0 (36.0–57.0)	23	0.11

*BMI* Body Mass Index, *DIC* disseminated intravascular coagulopathy, *SOFA* sequential organ failure assessment, *SAPS* simplified acute physiology score.

Values are reported as median and 25th–75th percentiles and as proportions.

*P value from Wilcoxon rank-sum and chi-squared test.

**ISTH 2011 overt classification (Taylor, F B et al. Thromb Haemost 86, no. 5 (November 1, 2001): 1327–30).

Ten (43.5%) and 11 (47.8%) patients received corticosteroid therapy (200 mg/day or more of hydrocortisone or equivalent dose of other glucocorticoids), in the control and treatment group, respectively (p = 0.77). No other immunomodulatory drugs were used during the study.

**Figure 1:**
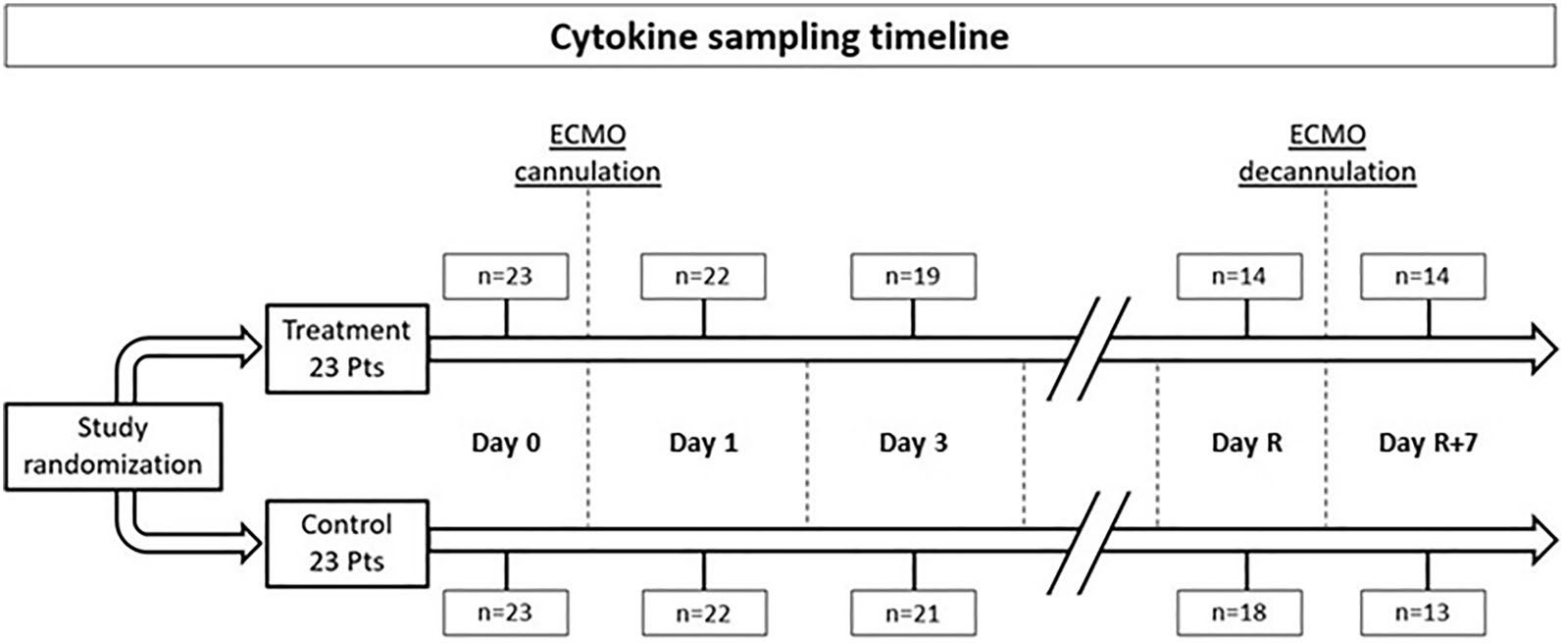
Cytokine sampling time during the study. Timepoints: Day 0 (prior to ECMO start), Day 1 (24 h after ECMO start), Day 3 (72 h after ECMO start), Day R (before ECMO removal), Day R + 7 (7 days after ECMO removal or before discharge from the ICU whichever happened first). *n* number of patients sampled.

All patients in both groups were sampled for IL-8, IL-6, IL-10, IL-1β, TNF-α but only 17 patients in the control group and 15 patients in the treatment group were sampled for Pro-ADM. As shown in Fig. 1, the number of sampled patients decreased during study days in both groups due either to clinical improvement or to premature discontinuation of the study. Circulating cytokines were slightly higher at baseline (at enrollment before initiation of ECMO, randomization and antithrombin administration) in the treatment compared to control group, this was especially noticeable for IL-1β and TNF-α (Table 2).

**Table 2:** Cytokines at enrollment in the study groups.

Cytokine	Reference Range	Control	N	Treatment	N	P value*
IL-8 (pg/mL)	< 40	23.0 (13.0–49.0)	23	45.0 (19.0–174.0)	23	0.11
IL-6 (pg/mL)	< 15	129.0 (37.0–392.0)	23	202.0 (63.0–909.0)	23	0.64
IL-10 (pg/mL)	< 15	134.0 (79.0–281.0)	23	220.0 (67.0–465.0)	23	0.30
IL-1β (pg/mL)	< 5	12.0 (9.0–21.0)	23	20.0 (11.0–33.0)	23	0.07
TNF-⍺ (pg/mL)	< 15	28.0 (24.0–45.0)	23	45.0 (31.0–78.0)	23	0.02
Pro-ADM (nmol/L)	< 0.55	2.1 (1.2–4.9)	17	2.4 (1.6–6.9)	15	0.43

Values are reported as median and 25^th^–75th percentiles. *N* Number of samples.

*P value from Wilcoxon rank-sum test.

**Figure 2:**
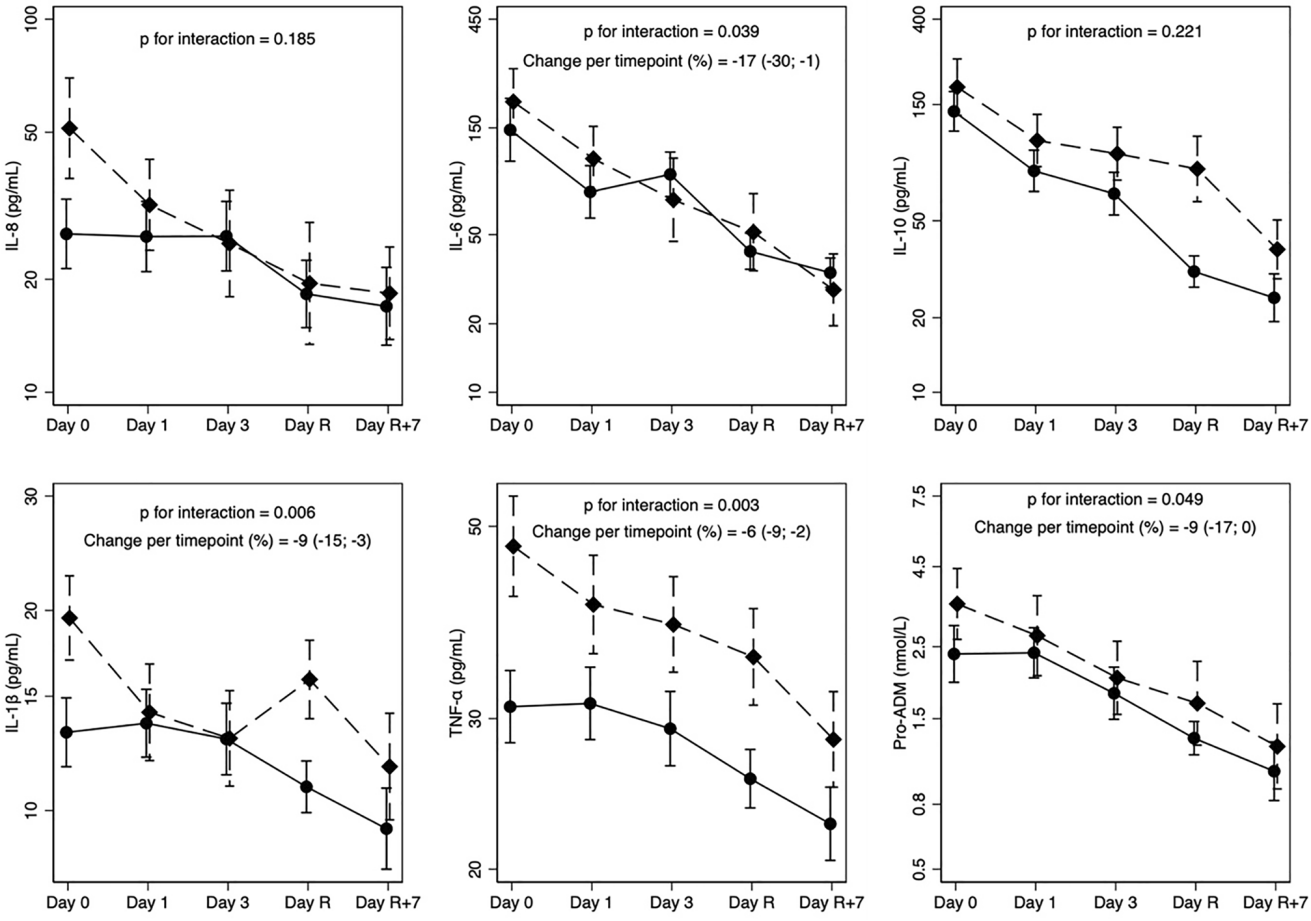
Cytokine trend in the study groups. Geometric means and standard error of the means of cytokines during the study. Day 0 (prior to ECMO start), Day 1 (24 h after ECMO start), Day 3 (72 h after ECMO start), Day R (before ECMO removal), Day R + 7 (7 days after ECMO removal or before discharge from the ICU whichever happened first). Dashed lines: control group, solid line: treatment group. P values for interaction are from models who treated time (timepoints) as a quantitative variable (i.e., we assigned values from 0 to 4 to the five timepoints).

Table 3 shows measured levels of cytokines in plasma during the study. Cytokines were not appreciably different in the two groups and in general markedly decreased over time (compare with Table 2). Looking at the five timepoints separately (Fig. 2), decreasing trends in both groups are evident. Moreover, cytokines reduction was stronger in the treatment group for IL-8, IL-6, TNF-α, and Pro-ADM, as indicated by converging trends. For IL-1β we noted irregular trends, but with stronger reduction in the treatment group (compare final and baseline values). Finally, a stronger reduction in the control group was observed for IL-10. Testing the interaction between the study group and timepoints treated as quantitative (one product term only) in general confirmed the visual impression: the administration of antithrombin led to a more rapid decrease (percent change per timepoint) over time of IL-6 (change − 17%, CI − 30%; − 1%), IL-1β (change − 9%, CI − 15%; − 3%), TNF-α (change − 6%, CI − 9%; − 2%), and Pro-ADM (change − 9%, CI − 17%; 0%). Conversely, the sensitivity analysis conducted treating timepoints as a categorical variable (four interaction terms) showed that formally only IL-1beta had a more rapid decrease over time (p for interaction = 0.023). Cytokines trends for each patient in the two groups are represented in Supplementary Fig. 1.

**Table 3:** Overall cytokines in the study groups during the study.

Cytokines	Reference range	Control	N	Treatment	N	P value*
IL-8 (pg/mL)	< 40	23.2 (2.6)	97	29.2 (4.0)	92	0.37
IL-6 (pg/mL)	< 15	74.6 (3.4)	97	85.0 (5.4)	93	0.26
IL-10 (pg/mL)	< 15	62.1 (2.8)	97	96.4 (3.4)	93	0.13
IL-1β (pg/mL)	< 5	12.1 (1.7)	97	14.9 (2.0)	93	0.51
TNF-⍺ (pg/mL)	< 15	28.3 (1.5)	97	38.7 (1.8)	93	0.18
Pro-ADM (nmol/L)	< 0.55	1.8 (2.1)	71	2.2 (2.7)	61	0.34

Values are reported as geometric means and geometric standard deviations (in brackets). *N* Number of samples.

*P value from random-effects linear regression models on log-transformed outcomes (cytokines), adjusted for basal value of dependent variable and time treated as a quantitative variable (i.e., we assigned values from 0 to 4 to the five timepoints).

Median duration of ECMO was 9.9 (3.9–16.9) days in the control group and 8.0 (5.2–13.1) days in the treatment group (p = 0.49). Median number of ECMO circuits exchanged was 1 (1–2) in both groups (p = 0.12). Eight patients died, 4 in the control and 4 in the treatment group respectively, six patients in the treatment group and four patients in the control group interrupted the study before the end of ECMO. The most common cause of premature discontinuation was intracranial hemorrhage. Except for IL-6 and IL-1β, all other cytokines were higher in patients who died compared to survivors (Supplementary Table 1 and Fig. 2).

**Figure 3:**
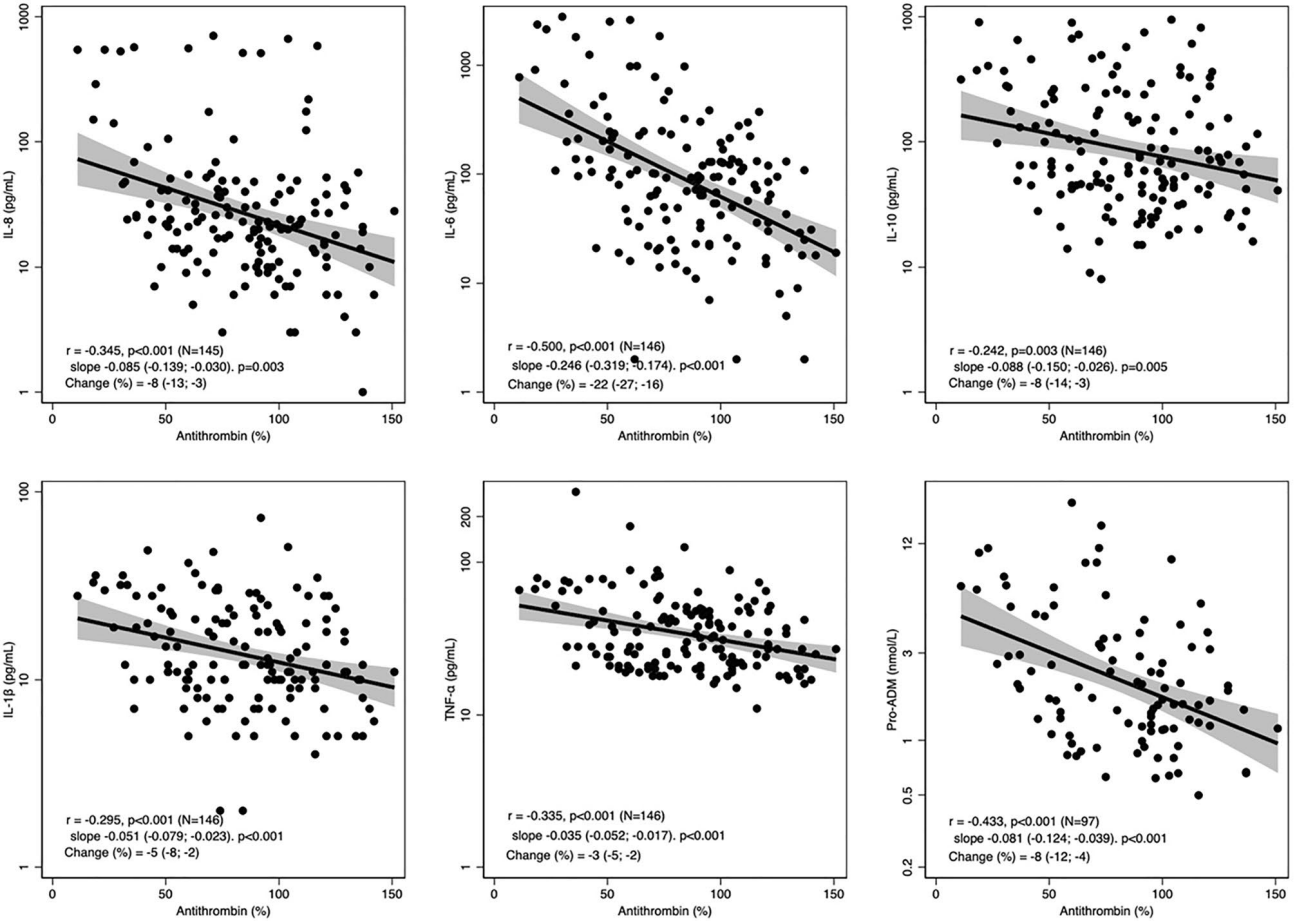
Correlation between cytokines and antithrombin. Pearson’s correlation coefficients, regression slopes and percent change in cytokine for 10% increase in antithrombin plasma level (%) with 95% CI from random-intercept linear regression models.

Interestingly, considering all timepoints, antithrombin was consistently associated with a reduction of cytokines with a strength of the correlation between weak to moderate^23^ as displayed in Fig. 3, and was not influenced by the administration of antithrombin (Supplementary Fig. 3).

## Discussion

In this study, we aimed at assessing the effect of antithrombin administration on the inflammatory response in patients undergoing veno-venous ECMO for severe respiratory failure. The main findings of our study are that firstly, inflammation consistently decreased during ECMO. Second, although patients supplemented with antithrombin had similar circulating levels of inflammatory mediators compared to controls, there was an trend towards a more rapid decrease in four pro-inflammatory cytokines: IL-6, IL-1β, TNF-α and Pro-ADM in patients who received antithrombin. Third, circulating levels of inflammatory mediators were negatively correlated with antithrombin levels. Taken together, our data might suggest that antithrombin plays a role in reducing inflammation that is possibly independent from its potentiating effect on anticoagulation^17^.

The inflammatory response has a central role in patients undergoing veno-venous ECMO. ARDS, which was by far the main cause of respiratory failure in the study population, is characterized by persistent elevation in local and systemic levels of cytokines (namely IL-1β, TNF-α, IL-6, and IL-8), which contribute to the progression of lung injury and to extrapulmonary organ dysfunction^24^. Down-regulation of systemic inflammation might be essential to restore homeostasis, to control the evolution of disease, and thus to improve clinical outcome. Moreover, dampening the inflammatory reaction might be particularly beneficial during ECMO, since the extracorporeal support itself triggers superimposed innate immune activation and inflammation^2^ based on the complex interaction between inflammation and coagulation. A rise in plasma concentrations of various pro-inflammatory cytokines, including TNF-α, IL-8, IL-1β, IL-6, has been demonstrated early after ECMO initiation^5^. Moreover, inflammation triggers a hypercoagulable state possibly resulting in heparin resistance^25^.

We found that the decrease of cytokines over time was consistent both in the control and treated patients. This could have different explanations mainly linked to the improvement of clinical conditions, response to antibiotics, better oxygenation, and splanchnic perfusion. Interestingly, the analysis suggested a trend towards faster decrease of some cytokines in the study group compared to the control group, indicating that supplementation of antithrombin to maintain a plasmatic level of 80–120% might have somehow played a role in reducing inflammation. However, it should be acknowledged that the sensitivity analysis conducted treating timepoints as categorical instead of continuous, replicated these results only for IL-1β. This “apparently anti-inflammatory” effect might be independent from the anticoagulant action, as the interaction of antithrombin with leukocytes and endothelial cells likely contributes to its anti-inflammatory properties. Other antithrombin effects include the induction of prostacyclin release, downregulation of P-selectin activity and prevention of leukocyte activation, which all result in decreased levels of pro-inflammatory cytokines^14^. Why this trend was primarily observed for IL-6, IL-1β, TNF-α and Pro-ADM remains to be elucidated.

We also observed that all cytokines except IL-1β, were consistently higher in patients who died compared to survivors. This confirms the findings of previous studies reporting that IL-6, IL-8 and TNF-α were associated with increased risk of mortality in patients on ECMO^26,27^.

We found a negative correlation between level of antithrombin and concentrations of all the measured cytokines. This correlation might result from different phenomena: on the one hand, higher levels of antithrombin may inhibit the production of cytokines due to its pleiotropic activities; on the other hand, patients with lower inflammation might have a greater endogenous production of antithrombin and a higher response to exogenous administration^28^. The inverse relationship between antithrombin and cytokine levels did not appear to be an effect of antithrombin administration, as it existed also in the control group.

This study has limitations. First, this was an ancillary study of a randomized controlled trial, and the sample size of the trial was not calculated for the endpoints of this study, also the amount of collected data decreased along the study in both study groups mainly due to premature interruption of the study. Second, by chance, patients in the control group presented with a higher level of inflammation and this may have influenced our results, even if we adjusted for this confounding factor in the analysis. Third, as shown in the main study^17^, antithrombin deficiency was not common in the study population and the control group showed quite high average values of antithrombin activity or tended to normalize along the ECMO course in patients who started with low basal values even without supplementation. Both these factors might affect the results, limiting the effect of antithrombin supplementation on the plasma cytokine levels. Fourth, the study population was heterogeneous in terms of etiology of respiratory failure, mostly enrolled in one of the two participating centers, and due to the small sample size, many confounders (or effect modifiers) could not be considered, including specific treatments which might impact on inflammation (i.e., steroids). Finally, higher doses of antithrombin might be required to fully exploit its anti-inflammatory properties^29,30^.

## Conclusions

In this study, a causal effect on reduction of inflammation by routine antithrombin supplementation during ECMO could not be demonstrated. However, there were indications of more rapid decrease of inflammatory mediators in patients who received antithrombin supplementation to maintain a plasmatic value of 80–120% compared to patients who were not supplemented. Also, the plasmatic level of antithrombin was correlated with the levels of cytokines, which suggests that antithrombin might have anti-inflammatory properties in patients supported with veno-venous ECMO. A larger and appropriately powered study is required to answer the question whether antithrombin administration reduces inflammation during veno-venous ECMO and to test the impact of this intervention on clinical outcomes.

## Supplementary Information

Supplementary Figure 1.

Supplementary Figure 2.

Supplementary Figure 3.

Supplementary Table 1.

## Data Availability

Collected data are available online at a dedicated website (https://ecmostudy.fbk.eu), with protected individual access for each participating center. The datasets used and/or analyzed during the current study are available from the corresponding author on reasonable request.

## References

[1] Lequier L, Horton SB, McMullan DM (2013). Extracorporeal membrane oxygenation circuitry. Pediatr. Crit. Care Med..

[2] Millar JE, Fanning JP, McDonald CI (2016). The inflammatory response to extracorporeal membrane oxygenation (ECMO): A review of the pathophysiology. Crit. Care Lond. Engl..

[3] Graulich J, Walzog B, Marcinkowski M (2000). Leukocyte and endothelial activation in a laboratory model of extracorporeal membrane oxygenation (ECMO). Pediatr. Res..

[4] Fortenberry JD, Bhardwaj V, Niemer P (1996). Neutrophil and cytokine activation with neonatal extracorporeal membrane oxygenation. J. Pediatr..

[5] McILwain RB, Timpa JG, Kurundkar AR (2010). Plasma concentrations of inflammatory cytokines rise rapidly during ECMO-related SIRS due to the release of preformed stores in the intestine. Lab. Invest..

[6] Mildner RJ, Taub N, Vyas JR (2005). Cytokine imbalance in infants receiving extracorporeal membrane oxygenation for respiratory failure. Biol. Neonate.

[7] Shen J, Yu W, Shi J (2013). Effect of venovenous extracorporeal membrane oxygenation on the heart in a healthy piglet model. J. Cardiothorac. Surg..

[8] Protti A, Iapichino GE, Di Nardo M (2019). Anticoagulation management and antithrombin supplementation practice during veno-venous extracorporeal membrane oxygenation: A worldwide survey. Anesthesiology.

[9] Roemisch J, Gray E, Hoffmann JN (2002). Antithrombin: a new look at the actions of a serine protease inhibitor. Blood Coagul. Fibrinol. Int. J. Haemost. Thromb..

[10] Schlömmer C, Brandtner A, Bachler M (2021). Antithrombin and its role in host defense and inflammation. Int. J. Mol. Sci..

[11] Uchiba M, Okajima K, Murakami K (1995). Effects of antithrombin III (AT III) and Trp49-modified AT III on plasma level of 6-keto-PGF1 alpha in rats. Thromb. Res..

[12] Yamashiro K, Kiryu J, Tsujikawa A (2001). Inhibitory effects of antithrombin III against leukocyte rolling and infiltration during endotoxin-induced uveitis in rats. Invest. Ophthalmol. Vis. Sci..

[13] Ostrovsky L, Woodman RC, Payne D (1997). Antithrombin III prevents and rapidly reverses leukocyte recruitment in ischemia/reperfusion. Circulation.

[14] Levy JH, Sniecinski RM, Welsby IJ (2015). Antithrombin: anti-inflammatory properties and clinical applications. Thromb. Haemost..

[15] Wang J, Wang Y, Wang J (2013). Antithrombin is protective against myocardial ischemia and reperfusion injury. J. Thromb. Haemost..

[16] Isik S, Tuncyurek P, Zengin NI (2012). Antithrombin prevents apoptosis by regulating inflammation in the liver in a model of cold ischemia/warm reperfusion injury. Hepatogastroenterology.

[17] Panigada M, Cucino A, Spinelli E (2020). A randomized controlled trial of antithrombin supplementation during extracorporeal membrane oxygenation. Crit. Care Med..

[18] Panigada M, Spinelli E, Cucino A (2019). Antithrombin supplementation during extracorporeal membrane oxygenation: Study protocol for a pilot randomized clinical trial. Trials.

[19] Nelson KM, Hansen LA, Steiner ME (2017). Continuous antithrombin III administration in pediatric veno-arterial extracorporeal membrane oxygenation. J. Pediatr. Pharmacol. Ther..

[20] Taylor FB, Toh CH, Hoots WK (2001). Towards definition, clinical and laboratory criteria, and a scoring system for disseminated intravascular coagulation. Thromb. Haemost..

[21] Siddiqui S, Gurung RL, Liu S (2019). Genetic polymorphisms and cytokine profile of different ethnicities inseptic shock patients and their association with mortality. Indian J. Crit. Care Med. Peer Rev..

[22] Tbahriti HF, Meknassi D, Moussaoui R (2013). Inflammatory status in chronic renal failure: The role of homocysteinemia and pro-inflammatory cytokines. World J. Nephrol..

[23] Evans JD (1996). Straightforward Statistics for the Behavioral Sciences.

[24] Meduri GU, Annane D, Chrousos GP (2009). Activation and regulation of systemic inflammation in ARDS. Chest.

[25] Levy JH, Connors JM (2021). Heparin resistance: Clinical perspectives and management strategies. N. Engl. J. Med..

[26] Risnes I, Wagner K, Ueland T (2008). Interleukin-6 may predict survival in extracorporeal membrane oxygenation treatment. Perfusion.

[27] Burrell AJC, Lubnow M, Enger TB (2017). The impact of venovenous extracorporeal membrane oxygenation on cytokine levels in patients with severe acute respiratory distress syndrome: A prospective, observational study. Crit. Care Resusc. J. Australas Acad. Crit. Care Med..

[28] Hayakawa M, Sawamura A, Yanagida Y (2008). The response of antithrombin III activity after supplementation decreases in proportion to the severity of sepsis and liver dysfunction. Shock.

[29] Warren BL, Eid A, Singer P (2001). High-dose antithrombin III in severe sepsis: A randomized controlled trial. JAMA.

[30] Inthorn D, Hoffmann JN, Hartl WH (1998). Effect of antithrombin III supplementation on inflammatory response in patients with severe sepsis. Shock.

