# Supplementary material for: The relationship between antithrombin administration and inflammation during veno-venous ECMO
Source: Sci Rep. 2022 Aug 22;12:14284. doi: 10.1038/s41598-022-17227-7 (PMC9395326; doi:10.1038/s41598-022-17227-7)
Supplement: Supplementary file 2 — Supplementary Figure 2. [file 41598_2022_17227_MOESM2_ESM.docx]

**Figure 2. Supplementary material**

Trend of cytokines in survivors and non-survivors


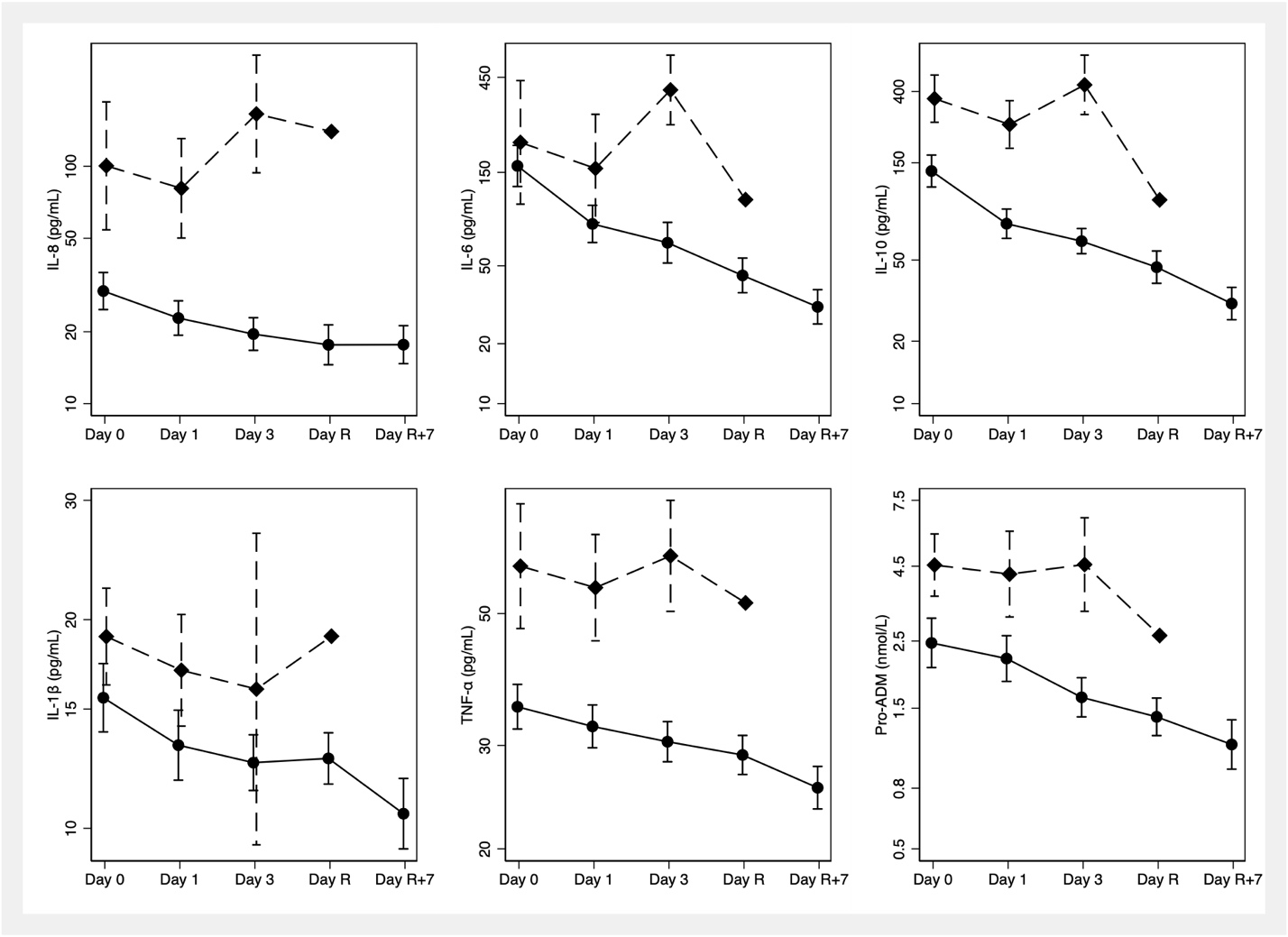


Geometric means and standard error of the means of cytokines during the study in survivors (solid lines) and non-survivors (dashed lines).

Day 0 (prior to ECMO start), Day 1 (24 h after ECMO start), Day 3 (72 h after ECMO start), Day R (before ECMO removal), Day R+7 (7 days after ECMO removal or before discharge from the ICU whichever happened first).
