# Supplementary material for: The relationship between antithrombin administration and inflammation during veno-venous ECMO
Source: Sci Rep. 2022 Aug 22;12:14284. doi: 10.1038/s41598-022-17227-7 (PMC9395326; doi:10.1038/s41598-022-17227-7)
Supplement: Supplementary file 3 — Supplementary Figure 3. [file 41598_2022_17227_MOESM3_ESM.docx]

**Figure 3. Supplementary material**

Correlation between cytokines and antithrombin in the study groups


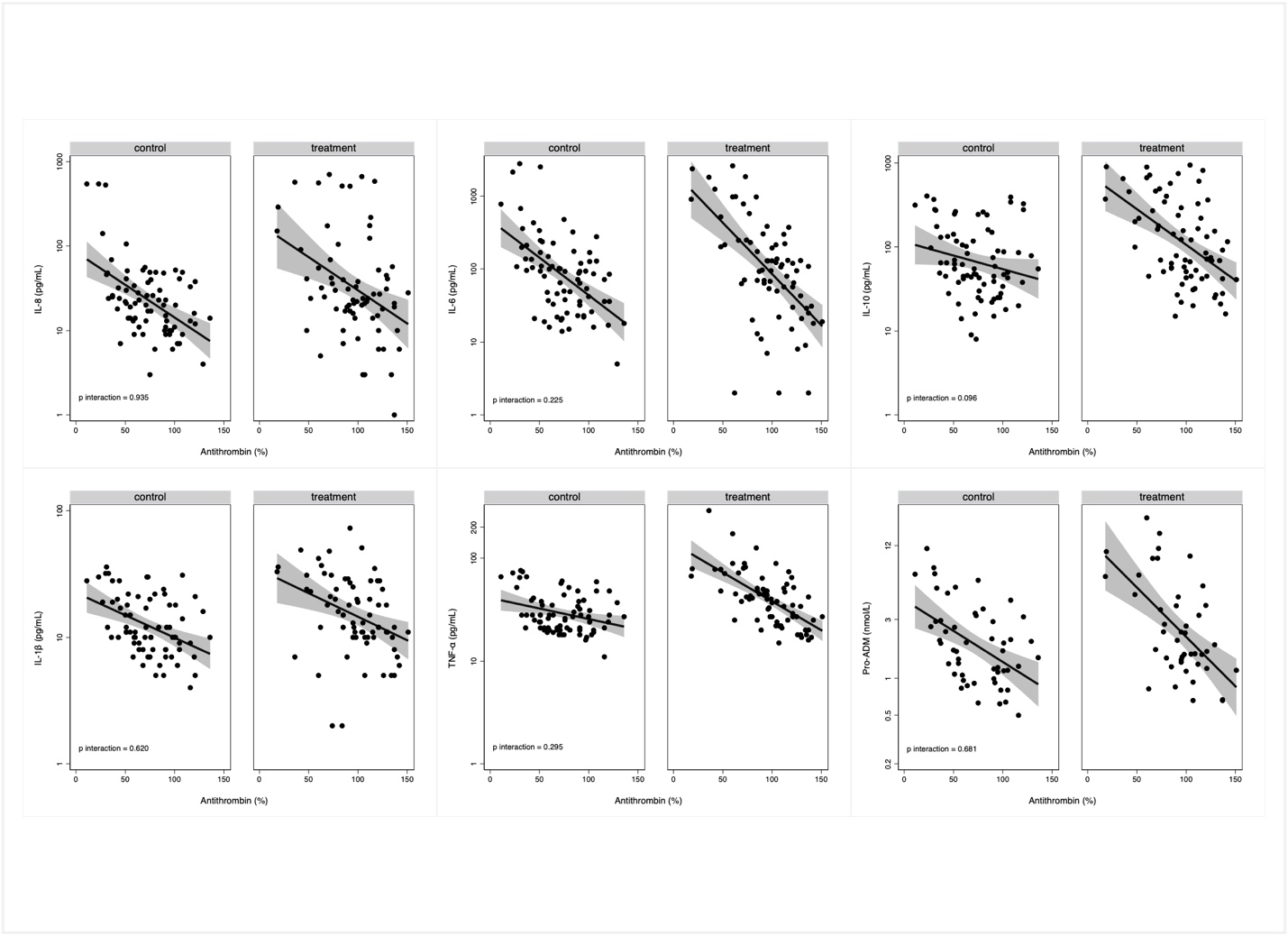


P value for interaction between the study groups and the plasmatic activity of antithrombin
