# Supplementary material for: The relationship between antithrombin administration and inflammation during veno-venous ECMO
Source: Sci Rep. 2022 Aug 22;12:14284. doi: 10.1038/s41598-022-17227-7 (PMC9395326; doi:10.1038/s41598-022-17227-7)
Supplement: Supplementary file 4 — Supplementary Table 1. [file 41598_2022_17227_MOESM4_ESM.docx]

**Table 1.** Supplementary material

Cytokines in survivors and non-survivors

| **Cytokine** | **Reference Range** | **Survivors** | **N** | **Non-survivors** | **N** | **P value*** |
| --- | --- | --- | --- | --- | --- | --- |
| IL-8 (pg/ml) | <40 | 21.5 (2.8) | 167 | 106.2 (4.2) | 22 | 0.000 |
| IL-6 (pg/ml) | <15 | 69.3 (4.1) | 167 | 216.0 (5.1) | 23 | 0.155 |
| IL-10 (pg/ml) | <15 | 65.1 (3.0) | 167 | 260.8 (2.1) | 23 | 0.001 |
| IL-1 β(pg/ml) | <5 | 12.9 (1.9) | 167 | 17.8 (1.9) | 23 | 0.312 |
| TNF-⍺ (pg/ml) | <15 | 30.6 (1.61) | 167 | 57.6 (1.7) | 23 | 0.002 |
| Pro-ADM (nmol/L) | <0.55 | 1.7 (2.3) | 112 | 4.2 (2.0) | 20 | 0.030 |

Values are reported as geometric means and geometric standard deviations (in brackets). N: number of samples.

*P value from random-effects linear models on log-transformed outcomes.
